# Cell Biological Responses after Shiga Toxin-1 Exposure to Primary Human Glomerular Microvascular Endothelial Cells from Pediatric and Adult Origin

**DOI:** 10.3390/ijms22115615

**Published:** 2021-05-25

**Authors:** Wouter J. C. Feitz, Petra A. van Setten, Thea J. A. M. van der Velden, Christoph Licht, Lambert P. J. W. van den Heuvel, Nicole C. A. J. van de Kar

**Affiliations:** 1 Department of Pediatric Nephrology, Amalia Children’s Hospital, Radboud Institute for Molecular Life Sciences, Radboudumc, 6525 GA Nijmegen, The Netherlands; Wouter.Feitz@radboudumc.nl (W.J.C.F.); Thea.vanderVelden@radboudumc.nl (T.J.A.M.v.d.V.); bert.vandenHeuvel@radboudumc.nl (L.P.J.W.v.d.H.); 2 Cell Biology Program, Research Institute, The Hospital for Sick Children, Toronto, ON M5G 1X8, Canada; christoph.licht@sickkids.ca; 3 Department of Pediatrics, Amalia Children’s Hospital, Radboudumc, 6525 GA Nijmegen, The Netherlands; petra.vansetten@radboudumc.nl; 4 Division of Nephrology, The Hospital for Sick Children, Toronto, ON M5G 1X8, Canada; 5 Department of Pediatrics, University of Toronto, Toronto, ON M5G 1X8, Canada; 6 Department of Development and Regeneration, Department of Pediatric Nephrology, KU, 3000 Leuven, Belgium

**Keywords:** hemolytic uremic syndrome, STEC-HUS, Shiga toxin, human glomerular microvascular endothelial cells from pediatric and adult origin

## Abstract

Hemolytic uremic syndrome (HUS) is characterized by a triad of symptoms consisting of hemolytic anemia, thrombocytopenia and acute renal failure. The most common form of HUS is caused by an infection with Shiga toxin (Stx) producing *Escherichia coli* bacteria (STEC-HUS), and the kidneys are the major organs affected. The development of HUS after an infection with Stx occurs most frequently in children under the age of 5 years. However, the cause for the higher incidence of STEC-HUS in children compared to adults is still not well understood. Human glomerular microvascular endothelial cells (HGMVECs) isolated and cultured from pediatric and adult kidney tissue were investigated with respect to Stx binding and different cellular responses. Shiga toxin-1 (Stx-1) inhibited protein synthesis in both pediatric and adult HGMVECs in a dose-dependent manner at basal conditions. The preincubation of pediatric and adult HGMVECs for 24 hrs with TNFα resulted in increased Stx binding to the cell surface and a 20–40% increase in protein synthesis inhibition in both age groups. A decreased proliferation of cells was found when a bromodeoxyuridine (BrdU) assay was performed. A trend towards a delay in endothelial wound closure was visible when pediatric and adult HGMVECs were incubated with Stx-1. Although minor differences between pediatric HGMVECs and adult HGMVECs were found in the assays applied in this study, no significant differences were observed. In conclusion, we have demonstrated that in vitro primary HGMVECs isolated from pediatric and adult kidneys do not significantly differ in their cell biological responses to Stx-1.

## 1. Introduction

Hemolytic uremic syndrome (HUS) is a thrombotic microangiopathy with hemolytic anemia, thrombocytopenia and the kidneys are the major organs affected. Infection with Shiga toxin (Stx) producing *Escherichia coli* bacteria (STEC) is one of the causes for the development of HUS (STEC-HUS) and is mostly seen in children under the age of 5 years [1]. The reason for a higher incidence of STEC-HUS in children compared to adults is still not understood.

Infection with STEC mainly occurs by ingestion of contaminated food products [2] leading to an acute, often bloody diarrhea. Approximately 10–15% of *E. coli* infected children will develop HUS [2]. After the transfer from the gut into the bloodstream, Stx interacts with cells by binding to its main receptor, globotriaosylceramide (Gb3) [3]. Uptake of the toxin into the cell results in the inhibition of protein synthesis and triggers apoptosis with cell death as result [4,5].

As Gb3 is the main functional receptor for Stx [3], it was hypothesized that the age-related incidence of HUS is caused by differential expression levels of Gb3 by the vascular endothelium of the glomerular capillaries. Studies have shown different levels of Gb3 in different parts of renal tissue with the highest levels present in the cortex [6], but an age-related difference has not been described yet.

In line with previous published work, we had the chance to isolate and culture primary human glomerular microvascular endothelial cells (HGMVECs) from pediatric kidneys. The interaction with Stx-1 was studied and examined in vitro [7] on primary HGMVECs isolated from two different pediatric kidneys (referred to as pediatric donor I and pediatric donor II) and from eight adult donors (referred to as adult donor I to adult donor VIII) with respect to binding, protein synthesis, cell proliferation and migration. 

## 2. Results

### 2.1. Stx-1 Binding to Pediatric and Adult Primary HGMVECs

**Figure 1:**
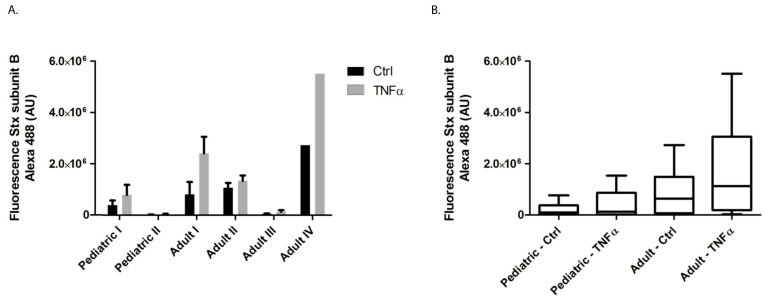
The binding of Alexa 488 labelled Stx-B on the endothelial cell surface of HGMVECs using flow cytometry. (**A**) Primary HGMVECs from two pediatric donors and four adult donors were incubated without (black bars) or with 10 ng/mL TNFα (grey bars) for 24 h. TNFα upregulated Stx-B binding in all six donors. (**B**) Results as shown in A expressed as pediatric vs. adult group. There was no significant difference between the pediatric vs. adult control groups or the pediatric vs. adult TNFα groups, in their ability to bind Alexa 488-labeled Stx-B (*p* = 0.145 for ctrl and *p* = 0.066 for TNFα by Mann−Whitney test). Mean values and SEM are given. Experiments were performed three times for pediatric donor I and II, adult donor I and III. Experiments were performed twice for adult donor II and once for adult donor IV.

To study the binding of Stx to the endothelial cell surface of primary cultured HGMVECs derived from two pediatric and four adult kidneys, flow cytometry analysis with the use of fluorescent labelled Stx-subunit B (Stx-B) was performed. This method is a reliable and sensitive method for the indirect detection of Gb3 [8]. The proinflammatory cytokine TNFα was used as positive control as it has been described to cause the upregulation of Gb3 [9]. In addition, TNFα is involved in the pathogenesis of STEC-HUS [10]. As shown in Figure 1, there was a high donor variation in terms of Stx-B binding on the cell surface of HGMVECs with the highest binding of Stx-B on HGMVECs derived from adult donor IV. Binding of Stx-B was low in untreated HGMVECs (control) derived from pediatric donor II and HGMVECs derived from adult donor III. Incubation of cells with 10 ng/mL of TNFα for 24 h resulted in an increase of Stx-B binding in all six donors with an average factor of 2.1 (factor 1.8 for pediatric HGMVECs and factor 2.3 for adult HGMVECs). Despite the results not being significantly different, Stx-B binding was less on the HGMVECs derived from pediatric tissue compared to the HGMVECs derived from adult tissue under basic conditions and after TNFα exposure (Figure 1B). 

### 2.2. Effect of Stx-1 on Protein Synthesis of Pediatric and Adult Primary HGMVECs

**Figure 2:**
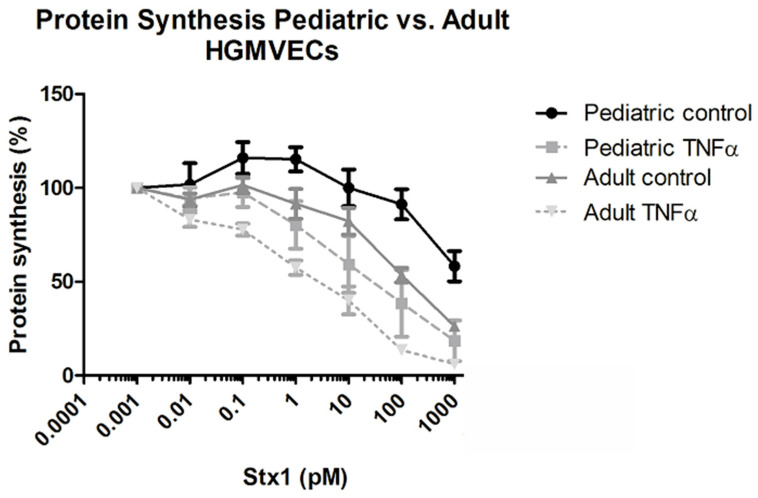
The effect of Stx-1 on the protein synthesis of primary HGMVECs measured by a ^3^H-leucine incorporation assay in pediatric HGMVECs from two donors and adult HGMVECs from three donors. Stx-1 at concentrations ranging from 0.001 pM to 1000 pM in control or cells preincubated with TNFα 10 ng/mL for 24 h were used. An increase of 20–40% in protein synthesis inhibition was measured when cells were preincubated with 10 ng/mL of TNFα for 24 h, however no significant difference by ANOVA between adult and pediatric conditions was found (*p* = 0.712). Mean values and SEM are given. Experiments were performed three times with HGMVECs from pediatric donor I and once with HGMVECs from pediatric donor II. Experiments were performed twice with HGMVECs from adult donor VII, once with adult donor V and once with adult donor VI.

The cytotoxic effect of Stx-1 on primary pediatric and adult HGMVECs was investigated by using a ^3^H-leucine incorporation assay/protein synthesis assay. Stx-1 concentrations were derived from experiments and results published in the past [7]. Stx-1 at concentrations ranging from 0.001 pM till 1000 pM affected protein synthesis in a concentration-dependent manner with a 20–40% increase in protein synthesis inhibition when cells were preincubated with 10 ng/mL of TNFα for 24 h (Figure 2). The protein synthesis of HGMVECs derived from two pediatric tissues (pediatric I and II) incubated with 1000 pM of Stx1 for 24 h decreased by 40% in the control group and with 80% in cells prestimulated with TNFα (Figure 2). In contrast, the protein synthesis of primary HGMVECs derived from three adult tissues (adult V, VI, VII) incubated with 1000 pM of Stx1 for 24 h decreased by 75% in the control group and 95% in cells prestimulated with TNFα (Figure 2). In summary, primary HGMVECs from adult kidney tissues showed a tendency towards a higher sensitivity for Stx-1 in terms of protein synthesis inhibition compared to HGMVECs derived from pediatric tissues. 

### 2.3. Effect of Stx-1 on the Proliferation of Pediatric and Adult HGMVECs

**Figure 3:**
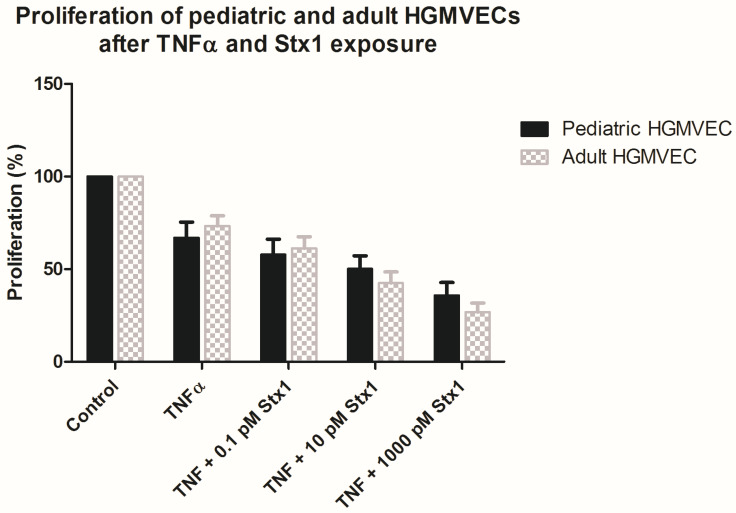
The effect of Stx-1 on the proliferation of HGMVECs by using a BrdU proliferation assay. Cells were incubated for 24 h with TNFα 10 ng/mL alone or preincubated with TNFα and incubated with 0.1, 10 and 1000 pM Stx-1. No significant difference by Mann−Whitney test was found between pediatric and adult HGMVECs for any single condition. Mean values and SEM are given. Experiments were executed in duplicate. Experiments were performed five times with HGMVECs from pediatric donor I and pediatric donor II. Experiments were performed five times with HGMVECs from adult donors III, three times with HGMVECs from adult donor I and twice with HGMVECs from adult donors II, IV and VIII.

The effect of Stx-1 on the proliferation of HGMVECs was studied using a BrdU proliferation assay. BrdU is incorporated into the cell during DNA synthesis in proliferating cells [11]. The proliferation of HGMVECs was investigated after the preincubation of cells with 10 ng/mL of TNFα and incubation of cells with 0.1 to 1000 pM Stx-1 for 24 h. As depicted in Figure 3, the proliferation pattern is similar in primary pediatric and adult HGMVECs. TNFα alone decreased cell proliferation by 27–33%, compared to control cells. Preincubation with 10 ng/mL of TNFα for 24 h and incubation with 0.1, 10 or 1000 pM of Stx-1 over a time period of 24 h decreased cell proliferation in a concentration-dependent manner. A concentration of 0.1 pM of Stx-1 with TNFα decreased cell proliferation by 38–42 % in both pediatric and adult HGMVECs, while a concentration of 1000 pM Stx-1 with TNFα decreased cell proliferation by 64% in pediatric HGMVECs and 73% in adult HGMVECs as compared with control cells. No significant difference in proliferation was established between pediatric and adult HGMVECs when statistics were applied for every single condition. However, a concentration of 10 pM Stx-1 or 1000 pM Stx-1 resulted in less proliferation of adult HGMVECs compared to pediatric HGMVECs. This is in line with the results of the protein synthesis assay.

### 2.4. Effect of Stx-1 on the Migration of Pediatric and Adult HGMVECs 

**Figure 4:**
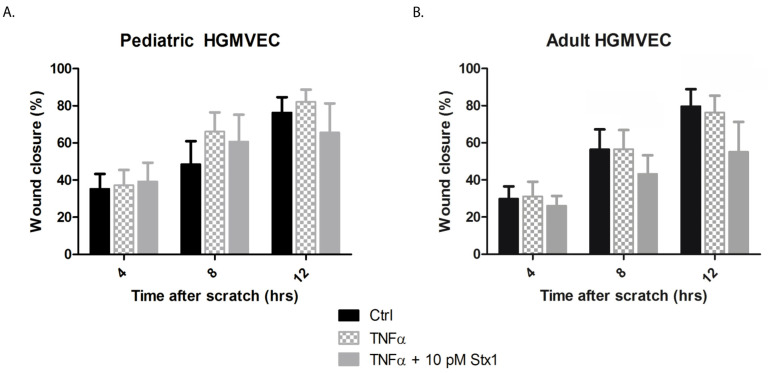
The effect of Stx-1 on wound closure of HGMVECs. (**A**) Primary pediatric HGMVECs or (**B**) adult HGMVECs were incubated with 10 ng/mL of TNFα or incubated with TNFα in combination with 10 pM Stx-1 for 12 h. A manual scratch wound was created, and images were taken after 0, 4, 8 and 12 h. No significant difference by Mann−Whitney test was calculated when the wound closure of pediatric HGMVECs was compared with the wound closure of adult HGMVECs, however a tendency towards a faster wound closure was measured when pediatric HGMVECs were used. Mean values and SEM are given. Experiments were performed three times with HGMVECs from pediatric donor I and donor II, three times with HGMVECs from adult donor I, twice with HGMVECs from adult donor III and once with HGMVECs from adult donor II and adult donor IV. Ctrl = control.

The effect of Stx-1 on the migration of HGMVECs was investigated by using an endothelial wound closure assay. A concentration of 10 pM Stx-1 was chosen as this concentration resulted in approximately 50% decreased protein synthesis in HGMVECs preincubated with TNFα 10 ng/mL as shown in Figure 2. TNFα alone had no significant effect on endothelial cell migration. A significant delay in wound closure was only measured after 8 h and 12 h of incubation between adult HGMVECs in the control group or preincubated with TNFα when compared with cells preincubated with TNFα and 10 pM Stx-1 (Figure 4). No significant difference was detected when the wound closures of pediatric and adult HGMVECs were compared. However, after 12 h of wound closure, TNFα in combination with 10 pM Stx-1 showed, although not significant, about 10% delay in wound closure when pediatric HMVECs were used and a significant 20% delay when adult HGMVECs were used and compared to their own control group. This is in line with the results found in the protein synthesis assay (Figure 2) and the results of the proliferation assay (Figure 3) where adult HGMVECs displayed a tendency to a higher sensitivity for Stx-1. 

## 3. Discussion

In this study, the interaction of Stx-1 with primary HGMVECs derived from pediatric and adult kidney tissue was examined by studying binding, protein synthesis, cell proliferation and migration. There was no difference between the binding of Stx-B to pediatric-derived HGMVECs as compared to adult HGMVECs. In both pediatric and adult HGMVECs, preincubation with TNFα led to increased Stx binding. Stx-1 was cytotoxic for HGMVECs in a dose-dependent manner, and TNFα increased the protein synthesis inhibition by 20–40%. Decreased proliferation of HGMVECs in a concentration-dependent manner was measured when cells were incubated with TNFα alone or when incubated with Stx-1 in combination with pretreatment with TNFα. There was no significant difference in pediatric cell migration after treatment with Stx-1, however a clear trend towards a delay in wound healing was visible. Even though an increased sensitivity of adult HGMVECs for Stx-1 compared to pediatric HGMVEC was seen at increasing concentrations of Stx-1, no significant differences were observed.

It was suggested that the glomerular endothelium of young children expresses higher levels of Gb3 compared to the glomerulus of adults. We have expanded on studies published in the past and used primary HGMVECs derived from two pediatric kidney tissues. Primary cells most closely represent the tissue of origin and are not genetically modified. They have not been used before to study Gb3 levels and primary pediatric cells are scarce, which make them a unique tool to use in the current examination.

Lingwood et al. compared the binding of Stx-B between the kidney tissue of infants and adults [6,12]. They found that Stx-B bound to the glomeruli of infants < 2 years and not to the glomeruli of adults [12]. It should be noted that, for the most part, kidney sections of steroid sensitive nephrotic syndrome (SSNS) patients were examined, which have been in contact with systemically and locally released cytokines which might result in higher levels of Gb3 and more Stx-B binding in different parts of the kidneys. In another publication from Boyd et al., they investigated the Gb3 content and Stx-B binding to human kidneys as a function of age [6]. Although they only had two samples of children and five samples of adults, the levels of Stx-B binding increased significantly in adult kidneys [6]. However, it must be noted that small amounts of a second Stx-binding glycolipid were detected in their experiments. This glycolipid terminated in the gal-α1-4gal structure. This structure is necessary for the binding of the toxin and thus may have played a role in the increments found [6]. Ergonul et al. compared frozen renal sections from subjects aged between 6 months to 85 years and showed that the pattern of Stx-1 binding was identical between the different age groups [13]. It is clear that we cannot compare our results of in vitro endothelial cell monoculture experiments with the above-mentioned pathology studies as they do not mimic the same cellular interactions and extracellular environment. 

The pediatric HGMVECS used in this study were isolated from kidneys of children under the age of 3 years, not suitable for transplantation, and are not representable for the glomerular endothelium of STEC-HUS patients. It is not feasible to obtain and culture HMGVECS from STEC-HUS patients as mostly no biopsy is clinically needed and not preferable, due to thrombocytopenia. However, it is possible to examine the host characteristics of STEC-HUS patients using blood outgrowth endothelial cells (BOECs) derived from pediatric patients with a history of STEC-HUS. BOECs are not derived from the kidneys of patients, nonetheless they do represent the endothelial characteristics and (epi)genetical background of the donor. In a recently published study, BOECs isolated from STEC-HUS patients showed no differences [14]. It is most likely that other factors, such as cytokines, chemokines, and circulating blood cells activated by the gastrointestinal STEC infection, activate the glomerular endothelial cells and their surrounding environment to a proinflammatory state. 

The variation in results upon Stx incubation between the cell cultures of various donors was most likely multifactorial and dependent on the expression of Gb3 on the cell surface. It is known that Gb3 expression at the cell surface can vary and depends on cell confluency as well as passages of primary cells used in the experiments. Subconfluent monolayers of HGMVECs as well as HUVECs show a higher expression of Gb3 on the surface leading to a higher cytotoxicity of Stx as compared to confluent monolayer of cells [15]. Van Setten et al. [7] showed that a confluent layer of HGMVECs only became susceptible for Stx-1 after preincubation with TNFα. Van Setten et al. [7] maintained cells for five days in a confluent state, while we treated the cells with Stx-1 the first day after reaching 80−100 % confluency. 

Blood cells as well as other glomerular cells are considered to play a role in the pathogenesis of disease. Ichimura [16] investigated bacterial responses under the influence of different concentrations of nitric oxide (NO). Their group suggested that nitric oxide generation in macrophages might stimulate the production of Stx. It has also been reported that Stx decreases the secretion of vascular endothelial growth factor (VEGF) [17]. VEGF is a potent angiogenic factor mainly produced by podocytes, that induces the formation of fenestrations in the endothelium of the glomerulus [18]. Decreased VEGF levels caused glomerular thrombotic microangiopathy (TMA) in mice [18]. Therefore, VEGF may play a role in the pathogenesis of STEC-HUS as HUS falls under the clinical picture of TMAs. 

Another pathway which may play a role in pathogenesis is the complement system, a system which is part of innate immunity. Overactivation of this system is known to be the main driver of disease in atypical HUS [19]. It has been shown that Stx binds factor H (FH), with delayed and reduced co-factor activity as result [20]. Furthermore, Stx caused downregulation of the complement inhibitor CD59 on tubular epithelial and glomerular endothelial cells [21] and from a clinical perspective, induced complement protein levels in patients with STEC-HUS at the time of hospital admission have been measured [22,23]. Among other publications, these studies suggest complement activation as a component of the pathophysiology of STEC-HUS. 

Still, at the moment, no model is available that recapitulates all the features of STEC infection and Gb3 expression is different in the kidneys from animals as compared to humans [24,25]. Future experiments using various kidney cells and/or kidney organoids in a co-culture system would bring us closer to the actual situation inside the body. Another arsenal available to study the pathophysiology of diseases is animal models. 

In conclusion, no differences in cell biological responses after Stx-1 exposure to primary HGMVECs from pediatric and adult origin were established. Other extrinsic or (epi)genetic factors might contribute to the sensitivity of the glomerular endothelium of children and the use of more sophisticated models, would help to gain a better understanding about the pathophysiology of this rare disease.

## 4. Materials and Methods

### 4.1. Ethics

This study was approved by the Medical Ethical Review Board of the Radboudumc, Nijmegen, The Netherlands. Written informed consent was obtained with a signature from parents/legal guardians or controls whose HGMVECs were used in this study. This study was executed in keeping with the regulations of the Declaration of Helsinki.

### 4.2. Isolation and Purification of Human Glomerular Microvascular Endothelial Cells (HGMVECs)

Studies were performed with human pediatric kidney tissue obtained from two kidneys of pediatric donors between the age of 2 and 3 years as well as eight healthy adult donors that were not suitable or disapproved for transplantation. The isolation and purification of the HGMVECs was carried out as previously described [7]. Briefly, glomeruli were isolated by dissecting the cortex followed by a gradual sieving procedure. Because the glomeruli of children are considerably smaller than those of adults, glomeruli were collected on top of smaller size screens (38, 53, 90 and 108 µm). Subsequently, glomeruli were digested with 0.1% (*w/v*) collagenase type 2 CLS (Worthington, NJ, USA) for 2 h at 37 °C. Glomerular remnants were resuspended in complete medium which consisted of M199 (Gibco Thermo Fisher Scientific, Waltham, MA, USA) supplemented with 10% heat inactivated newborn calf serum (Gibco Thermo Fisher Scientific, Waltham, MA, USA), 10% heat inactivated human serum (Innovative Research, Novi, MI), 2 mmol/L glutamine (Gibco Thermo Fisher Scientific, Waltham, MA, USA), 1% penicillin/streptomycin (Gibco Thermo Fisher Scientific, Waltham, MA, USA), 5 U/mL heparin (Leo Pharmaceuticals, Weesp, The Netherlands) and 150 mg/L of endothelial cell growth factor (extracted from bovine brains as described by Maciag et al. [26]) and plated on gelatin coated plates (Corning Incorporated, Kennebunk, ME, USA). Attachment occurred within one or two days after which endothelial and epithelial cells started to grow out with high proliferation rates. Primary outgrowths were selectively trypsinized and filtrated through a 38 µm sieve to enrich HGMVECs. HGMVECs were specifically collected by performing an immunomagnetic separation technique using a monoclonal antibody against platelet cell adhesion molecule-1 (PECAM-1) as an endothelial specific antibody. Highly purified populations of pediatric HGMVECs were obtained by repeating the immunomagnetic separation technique once or twice. Cells with 80–100 % confluency, passage 6–11, were used for experiments.

### 4.3. Reagents 

Shiga toxin type 1 (Stx-1) was ordered from Phoenix Lab (Tufts Medical Center, Boston, MA). The amount of LPS remaining in the preparations was determined as <0.01 endotoxin units per mL. Alexa 488 labeled Shiga toxin subunit B (Stx-B) was kindly provided by C. Lingwood, The Hospital for Sick Children, Toronto, ON, Canada. 

### 4.4. Flow Cytometry

HGMVECs were seeded in 24-well plates. Cells were incubated with or without 10 ng/mL of TNFα (Roche Diagnostics, Indianapolis, IN, USA) for 24 h. Next, cells were fixed with 0.5% PFA/PBS for 5 min at room temperature. Cells were incubated with Alexa 488-labeled Stx-B at a concentration of 5 ug/mL and stored for 30 min at 4 °C protected from light. Cells were trypsinized, suspended in 10% FBS/PBS, spun down by centrifugation and resuspended in 0.1% BSA/PBS. Read out was done with the use of a CytoFlex Flow Cytometer (Beckman Coulter, Brea, CA, USA). 

### 4.5. Protein Synthesis by Radiolabeled ^3^H-Leucine Incorporation Assay 

HGMVECs were seeded in 24-well plates. Cells were prestimulated with or without 10 ng/mL of TNFα for 24 h and incubated with increasing concentrations of Stx-1 in a range of 0.001−1000 pM for 24 h together with ^3^H-leucine (PerkinElmer, Boston, MA, USA). The ^3^H-leucine incorporation assay was further carried out as previously described [14]. 

### 4.6. Bromodeoxyuridine (BrdU) Proliferation Assay 

HGMVECs were seeded in 96-well plates and preincubated with 10 ng/mL TNFα for 24 h. Stx-1 in a concentration of 0.1−1000 pM in combination with 10 µM BrdU (Sigma-Aldrich, St. Louis, MO, USA) was added and stored in a 37 °C incubator with 5% CO_2_ for 24 h. The BrdU assay was performed according to manufacturer’s protocol (Sigma-Aldrich, St. Louis, MO, USA). 

### 4.7. Endothelial Wound Closure Assay

HGMVECs were seeded in 24-well plates and preincubated with 10 ng/mL TNFα for 24 h. After 24 h, fresh media with or without 10 pM Stx1 was added, and a manual scratch wound was created with a 200 uL pipette tip. Next, a previously published protocol was followed [14,27]. Images were captured after timepoint 0, 4, 8 and 12 h with a Zeiss Axiovert 25 inverted transmitted light microscope (Zeiss, Oberkochen, Baden-Württemberg, Germany) in combination with a Canon EOS 1000D camera (Canon, Tokio, Japan). Analysis of the images was performed with the use of ImageJ software version 1.51n (LCOI, Madison, WI, USA). 

### 4.8. Statistical Analysis

Data were analyzed by nonparametric Mann−Whitney test or Kruskal−Wallis test. A *p*-value of <0.05 was set as statistically significant. All statistical analysis were performed using GraphPad Prism version 5.03 (GraphPad Software, La Jolla, CA, USA) or SPSS (IBM, Amsterdam, Netherlands). Data are expressed as mean ± SEM. 

## Data Availability

Not applicable.

## References

[1] Mele C., Remuzzi G., Noris M. (2014). Hemolytic uremic syndrome. Semin. Immunopathol..

[2] Tarr P.I., Gordon C.A., Chandler W.L. (2005). Shiga-toxin-producing Escherichia coli and haemolytic uraemic syndrome. Lancet.

[3] Lingwood C. (2020). Verotoxin Receptor-Based Pathology and Therapies. Front. Cell. Infect. Microbiol..

[4] Karpman D., Loos S., Tati R., Arvidsson I. (2016). Haemolytic uraemic syndrome. J. Intern. Med..

[5] Pijpers A.H., Van Setten P., Heuvel L.P.V.D., Assmann K.J., Dijkman H.B., Pennings A.H., Monnens L., Van Hinsbergh V.W. (2001). Verocytotoxin-induced apoptosis of human microvascular endothelial cells. J. Am. Soc. Nephrol..

[6] Boyd B., Lingwood C. (1989). Verotoxin Receptor Glycolipid in Human Renal Tissue. Nephron.

[7] Van Setten P.A., van Hinsbergh V.W., van der Velden T.J., van de Kar N.C., Vermeer M., Mahan J.D., Assmann K.J., van den Heuvel L.P., Monnens L.A. (1997). Effects of TNF alpha on verocytotoxin cytotoxicity in purified human glomerular microvascular endothelial cells. Kidney Int..

[8] Kim M., Binnington B., Sakac D., Fernandes K.R., Shi S.P., Lingwood C.A., Branch D.R. (2011). Comparison of detection methods for cell surface globotriaosylceramide. J. Immunol. Methods.

[9] Van de Kar N.C., Monnens L.A., Karmali M.A., van Hinsbergh V.W. (1992). Tumor necrosis factor and interleukin-1 induce expression of the verocytotoxin receptor globotriaosylceramide on human endothelial cells: Implications for the pathogenesis of the hemolytic uremic syndrome. Blood.

[10] Exeni R.A., Fernandez-Brando R.J., Santiago A.P., Fiorentino G.A., Exeni A.M., Ramos M.V., Palermo M.S. (2018). Pathogenic role of inflammatory response during Shiga toxin-associated hemolytic uremic syndrome (HUS). Pediatr. Nephrol..

[11] Takeyoshi M., Yamasaki K., Yakabe Y., Takatsuki M., Kimber I. (2001). Development of non-radio isotopic endpoint of murine local lymph node assay based on 5-bromo-2’-deoxyuridine (BrdU) incorporation. Toxicol. Lett..

[12] Lingwood C. (1994). Verotoxin-Binding in Human Renal Sections. Nephron.

[13] Ergonul Z., Clayton F., Fogo A.B., Kohan D.E. (2003). Shigatoxin-1 binding and receptor expression in human kidneys do not change with age. Pediatr. Nephrol..

[14] Feitz W.J.C., Van De Kar N.C.A.J., Cheong I., Van Der Velden T.J.A.M., Ortiz-Sandoval C.G., Orth-Höller D., Heuvel L.P.J.W.V.D., Licht C. (2020). Primary Human Derived Blood Outgrowth Endothelial Cells: An Appropriate In Vitro Model to Study Shiga Toxin Mediated Damage of Endothelial Cells. Toxins.

[15] Obrig T.G., Del Vecchio P.J., Brown J., Moran T.P., Rowland B.M., Judge T.K., Rothman S.W. (1988). Direct cytotoxic action of Shiga toxin on human vascular endothelial cells. Infect. Immun..

[16] Ichimura K., Shimizu T., Matsumoto A., Hirai S., Yokoyama E., Takeuchi H., Yahiro K., Noda M. (2017). Nitric oxide-enhanced Shiga toxin production was regulated by Fur and RecA in enterohemorrhagic *Escherichia coli* O157. Microbiology.

[17] Psotka M.A., Obata F., Kolling G.L., Gross L.K., Saleem M.A., Satchell S.C., Mathieson P.W., Obrig T.G. (2009). Shiga Toxin 2 Targets the Murine Renal Collecting Duct Epithelium. Infect. Immun..

[18] Eremina V., Jefferson J.A., Kowalewska J., Hochster H., Haas M., Weisstuch J., Richardson C., Kopp J.B., Kabir M.G., Backx P.H. (2008). VEGF Inhibition and Renal Thrombotic Microangiopathy. N. Engl. J. Med..

[19] Conway E.M. (2015). HUS and the case for complement. Blood.

[20] Orth D., Khan A.B., Naim A., Grif K., Brockmeyer J., Karch H., Joannidis M., Clark S.J., Day A.J., Fidanzi S. (2009). Shiga Toxin Activates Complement and Binds Factor H: Evidence for an Active Role of Complement in Hemolytic Uremic Syndrome. J. Immunol..

[21] Ehrlenbach S., Rosales A., Posch W., Wilflingseder D., Hermann M., Brockmeyer J., Karch H., Satchell S.C., Würzner R., Orth-Höller D. (2013). Shiga Toxin 2 Reduces Complement Inhibitor CD59 Expression on Human Renal Tubular Epithelial and Glomerular Endothelial Cells. Infect. Immun..

[22] Monnens L., Molenaar J., Lambert P.H., Proesmans W., Van Munster P. (1980). The complement system in hemolytic-uremic syndrome in childhood. Clin. Nephrol..

[23] Thurman J.M., Marians R., Emlen W., Wood S., Smith C., Akana H., Holers V.M., Lesser M., Kline M., Hoffman C. (2009). Alternative Pathway of Complement in Children with Diarrhea-Associated Hemolytic Uremic Syndrome. Clin. J. Am. Soc. Nephrol..

[24] Jeong Y.-J., Park S.-K., Yoon S.-J., Park Y.J., Lee M.-S. (2018). Experimental In Vivo Models of Bacterial Shiga Toxin-Associated Hemolytic Uremic Syndrome. J. Microbiol. Biotechnol..

[25] Obrig T.G. (2010). Escherichia coli Shiga Toxin Mechanisms of Action in Renal Disease. Toxins.

[26] Maciag T., Cerundolo J., Ilsley S., Kelly P.R., Forand R. (1979). An endothelial cell growth factor from bovine hypothalamus: Identification and partial characterization. Proc. Natl. Acad. Sci. USA.

[27] Liang C.-C., Park A.Y., Guan J.-L. (2007). In vitro scratch assay: A convenient and inexpensive method for analysis of cell migration in vitro. Nat. Protoc..

